# Supplementary material for: Development of PET Radioisotope Copper-64-Labeled Theranostic Immunoliposomes for EGFR Overexpressing Cancer-Targeted Therapy and Imaging
Source: Int J Mol Sci. 2024 Feb 2;25(3):1813. doi: 10.3390/ijms25031813 (PMC10855797; doi:10.3390/ijms25031813)
Supplement: Supplementary file 1 [file ijms-25-01813-s001.zip › ijms-2846867-supplementary.pdf]

Supplementary Material

Development of PET radioisotope copper-64-labeled theranostic immunoliposomes for EGFR overexpressing cancer-targeted therapy and imaging

Hwa Yeon Jeong [1,2], Seong Jae Kang [1], Min Woo Kim [1], In ho Jeong [1], Moon Jung Choi [1], Cheulhee Jung [2], In Ho Song [3], Tae Sup Lee [3] and Yong Serk Park [1,*]

[1] Department of Biomedical Laboratory Science, Yonsei University, Wonju 26493, Republic of Korea;
[2] Department of Biotechnology, College of Life Sciences and Biotechnology, Korea University, Seoul 02841, Republic of Korea;
[3] Division of RI-Convergence Research, Korea Institute of Radiological and Medical Science, Seoul 01812, Republic of Korea.

* Correspondence: Yong Serk Park
Department of Biomedical Laboratory Science, Yonsei University, Wonju 26493, Republic of Korea
Tel +82 033 760 2448
Email parkys@yonsei.ac.kr

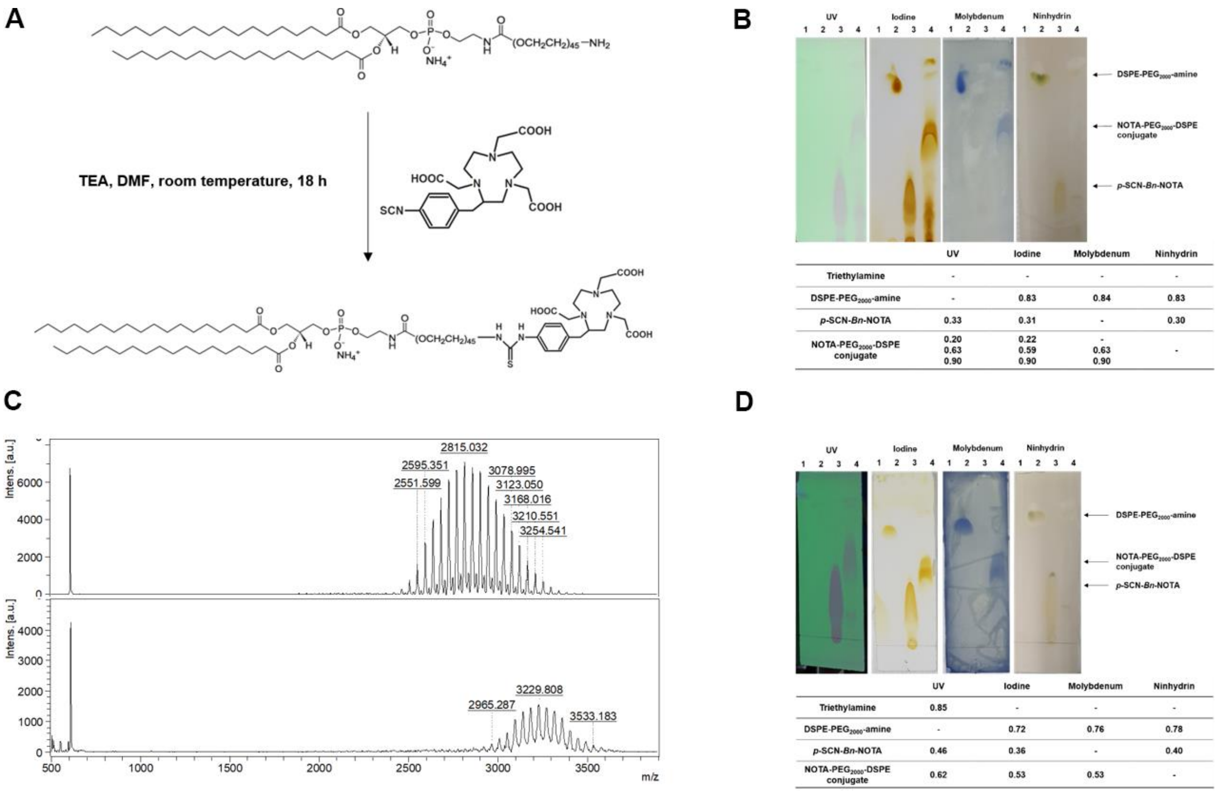

	UV	Iodine	Molybdenum	Ninhydrin
Triethylamine	-	-	-	-
DSPE-PEG$_{2000}$-amine	-	0.83	0.84	0.83
p-SCN-*Bn*-NOTA	0.33	0.31	-	0.30
NOTA-PEG$_{2000}$-DSPE conjugate	0.20<br>0.63<br>0.90	0.22<br>0.59<br>0.90	-<br>0.63<br>0.90	-

	UV	Iodine	Molybdenum	Ninhydrin
Triethylamine	0.85	-	-	-
DSPE-PEG$_{2000}$-amine	-	0.72	0.76	0.78
p-SCN-*Bn*-NOTA	0.46	0.36	-	0.40
NOTA-PEG$_{2000}$-DSPE conjugate	0.62	0.53	0.53	-

Supplementary Figure S1. Analysis of DSPE-PEG2000-NOTA conjugate. **(A)** DSPE-PEG2000-amine was conjugated with *p*-SCN-*Bn*-NOTA and **(B)** the conjugate was analyzed by a TLC plate. **(C)** MALDI-TOF mass spectrometric analysis of the purified DSPE-PEG2000-NOTA conjugate and DSPE-PEG2000-amine demonstrated an increase in mass from 2859.8 to 3249.1 after conjugation with the *p*-SCN-*Bn*-NOTA. This corresponds to 0.86 NOTA molecules conjugated to one DSPE-PEG2000-amine. The x-axis shows the mass/charge (m/z) values, and the y-axis the signal intensity. And **(D)** the purified DSPE-PEG2000-NOTA was confirmed with a TLC plate.

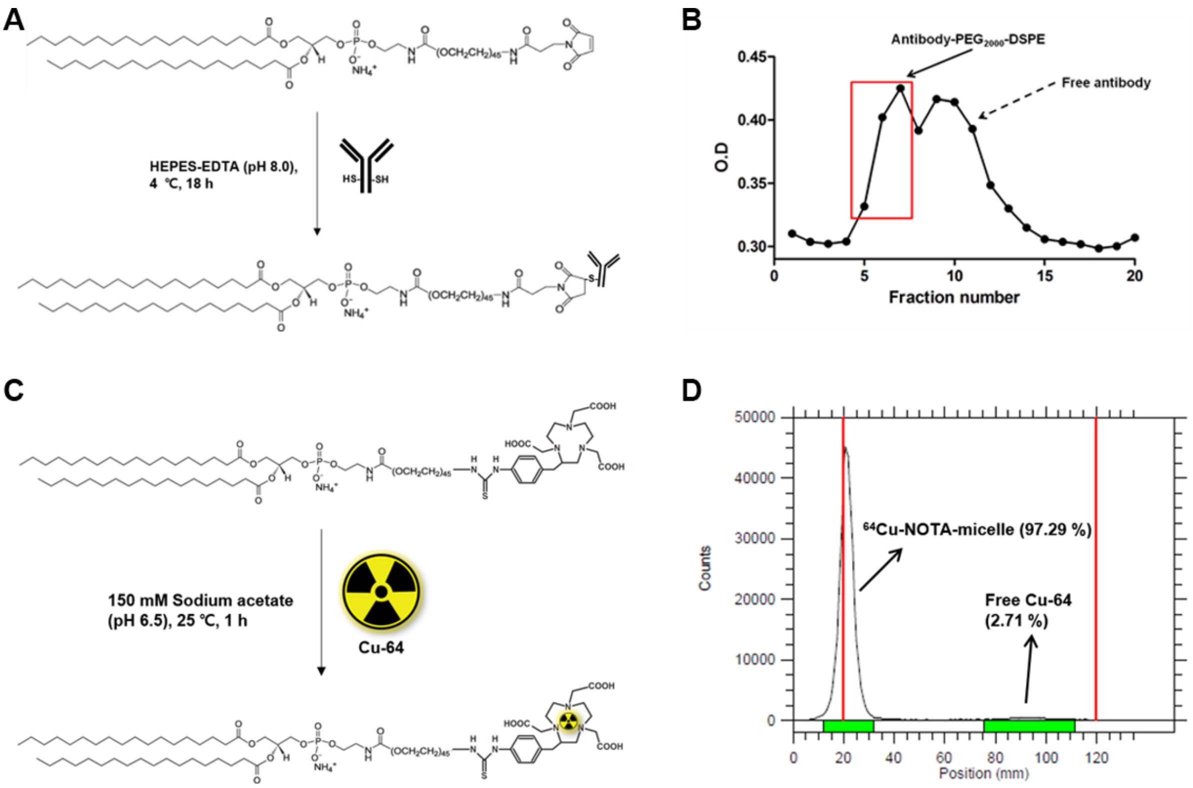

Supplementary Figure S2. Preparation of antibody-conjugated or copper-64-labeled micelles. **(A-B)** Anti-EGFR antibody (cetuximab) was conjugated to DSPE-PEG2000-maleimide in micelles, and the antibody conjugation was analyzed with a sepharose CL-4B column. **(C-D)** Copper-64 was labeled to DSPE-PEG2000-NOTA conjugate in micelles, and the copper-64-labeled micelles were analyzed with iTLC-SG.

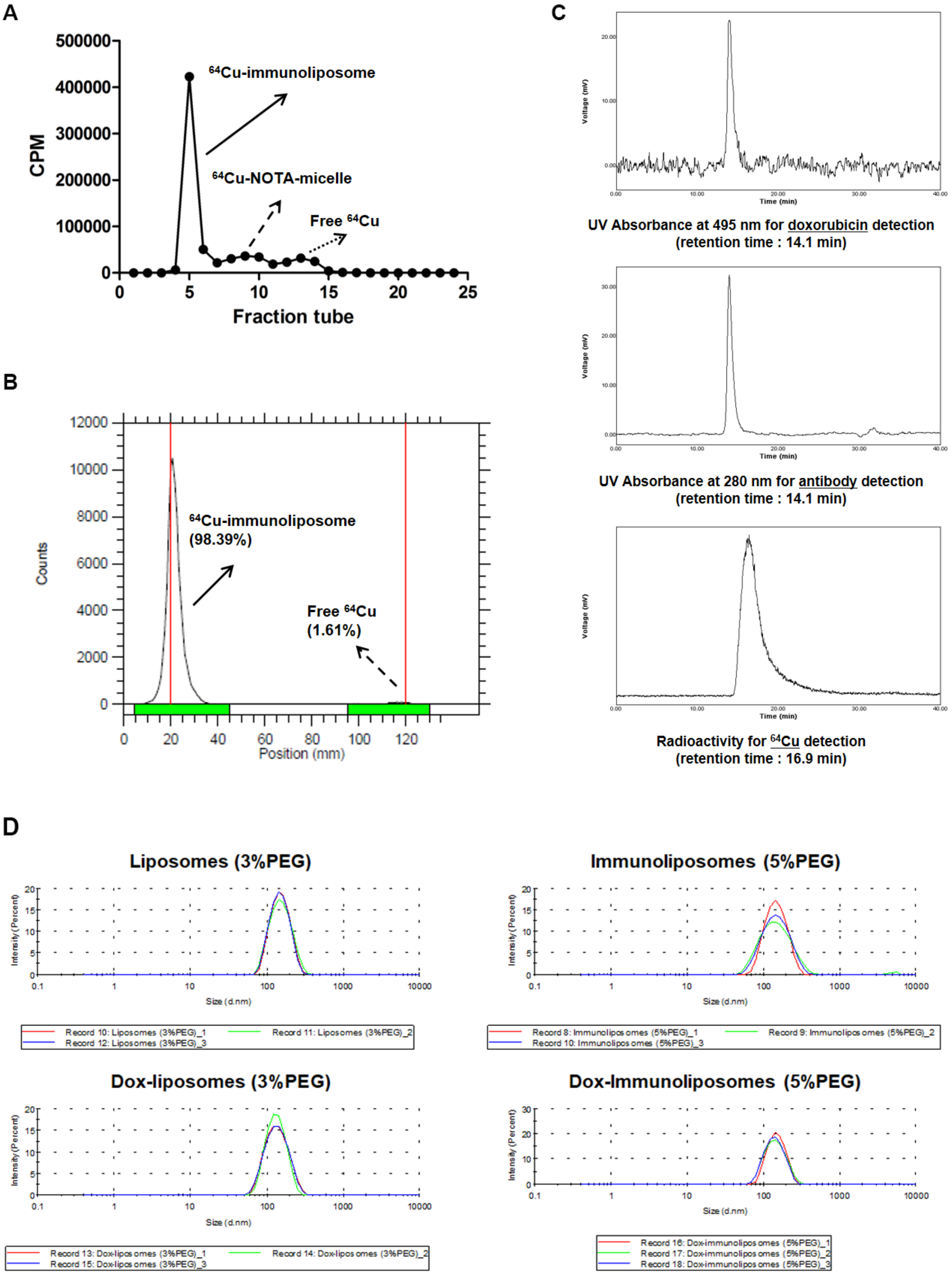

Supplementary Figure S3. Purification and evaluation of ^{64}Cu-Dox-immunoliposomes. ^{64}Cu-micelles were inserted into doxorubicin-encapsulating liposomes at 37°C for 4 h. **(A)** The ^{64}Cu-labeled Dox-immunoliposomes were purified with sepharose CL-4B column and then analyzed using gamma-counter. The ^{64}Cu-Dox-immunoliposomes in the 4th and 5th fractions from the column were analyzed with iTLC and HPLC. **(B)** ITLC analysis showed 98.0% or higher purity of the ^{64}Cu-Dox-immunoliposomes. **(C)** HPLC chromatograms of ^{64}Cu-Dox-immunoliposomes exhibited a retention time of 14.1 min, detected

by both doxorubicin detection at 495 nm and antibody detection at 280 nm. Meanwhile, the retention time measured with copper-64 was 16.9 min. (D) Size distribution by intensity of various liposomes in this study.

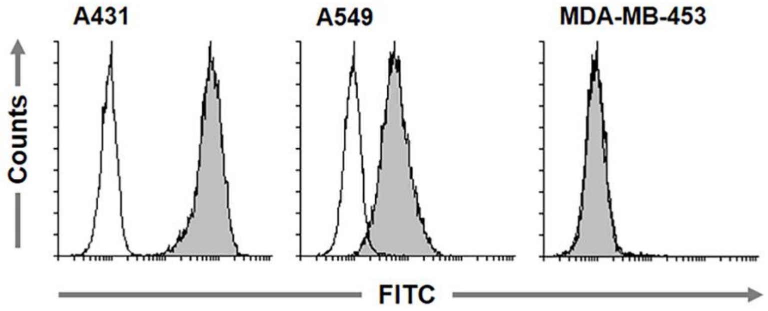

Supplementary Figure S4. Evaluation of EGF receptor expression on the cell surface. EGF receptor expression on the surface of A431, A549, and MDA-MB-453 cells was compared by flow cytometry.

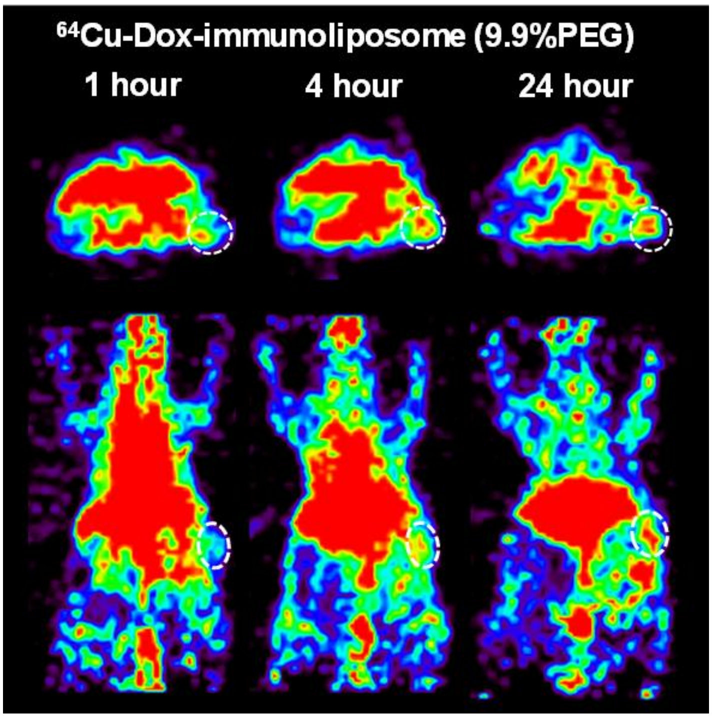

Supplementary Figure S5. Biodistribution of ^{64}Cu-Dox-immunoliposomes (10%PEG) in xenografted A549 tumor models. PET imaging of the A549 tumor-bearing mice at 1 h, 4 h, and 24 h after ^{64}Cu-Dox-immunoliposomes (10%PEG) administration.

Supplementary Table S1. Tissue uptakes of ^{64}Cu-PCTA-Cetuximab

	^{64}Cu-PCTA-Cetuximab
	**48 h**
**Blood**	9.7 ± 0.4
**Heart**	3.4 ± 0.2
**Liver**	10.4 ± 0.6
**Lung**	6.8 ± 0.7
**Spleen**	6.8 ± 0.5
**Kidney**	5.0 ± 0.3
**Muscle**	1.9 ± 0.3
**Tumor**	9.2 ± 1.1
**T/B**	1.0 ± 0.1
**T/M**	4.9 ± 0.8
**T/L**	0.9 ± 0.1

Notes: Tissue uptake of ^{64}Cu-PCTA-Cetuximab in A549 tumor xenograft-bearing BALB/c-nude mice (n = 3) at 48 h post-i.v. injection expressed as %ID/g; Data (%ID/g) were presented as mean ± S.D. (n = 3).

Supplementary Table S2. Physicochemical properties of liposomes

Nanoparticles	Size (nm)*	Zeta-potential (mV)*	Polydisp-ersity Index	Doxorubicin Encapsulation Efficiency (%)
**Dox-immunoliposomes (5%PEG)**	147.5 ± 4.0**	-2.4 ± 0.6**	0.06	96.1
**Dox-liposomes (8%PEG)**	134.5 ± 1.3	-3.2 ± 0.5	0.08	82.8
**Dox-immunoliposomes (10%PEG)**	132.7 ± 4.8	-6.2 ± 0.2	0.16	79.8

Notes: *The particle size and zeta-potentials were measured 3 times using a zeta sizer. **The particle size (nm); average particle size ± S.D. Zeta potential (mV); average zeta-potential ± S.D.

Abbreviations: PEG, polyethylene glycol.

Supplementary Table S3. Biodistribution of ^{64}Cu-Dox-immunoliposomes containing varied amounts of PEG

	^{64}Cu-Dox-immunoliposome (5%PEG)			^{64}Cu-Dox-immunoliposome (10%PEG)		
	**1 h**	**4 h**	**24 h**	**1 h**	**4 h**	**24 h**
**Blood**	26.4 ± 3.2	24.1 ± 6.4	3.1 ± 0.4	32.0 ± 5.7	28.5 ± 3.1	5.3 ± 1.9
**Heart**	3.3 ± 0.1	2.8 ± 0.7	0.9 ± 0.0	3.8 ± 0.4	3.5 ± 0.2	1.3 ± 0.3
**Liver**	11.4 ± 3.2	19.7 ± 0.9	17.8 ± 4.3	11.0 ± 3.8	16.0 ± 1.8	16.6 ± 1.4
**Lung**	7.4 ± 1.5	7.7 ± 1.8	2.0 ± 0.2	9.7 ± 4.1	8.6 ± 1.0	2.5 ± 0.5
**Spleen**	21.6 ± 2.9	44.3 ± 8.8	80.9 ± 34.7	28.1 ± 7.2	45.5 ± 12.1	87.5 ± 22.6
**Kidney**	6.6 ± 0.8	6.0 ± 1.1	3.3 ± 0.7	6.1 ± 1.0	7.8 ± 1.1	4.6 ± 0.4
**Muscle**	0.7 ± 0.1	0.8 ± 0.0	0.4 ± 0.1	0.7 ± 0.1	1.0 ± 0.1	0.6 ± 0.1
**Tumor**	1.4 ± 0.4	2.9 ± 0.5	2.8 ± 0.0	1.3 ± 0.6	2.7 ± 0.4	4.0 ± 0.4
**T/B**	0.1 ± 0.0	0.1 ± 0.0	0.9 ± 0.1	0.0	0.1 ± 0.0	0.8 ± 0.3
**T/M**	2.1 ± 0.7	3.7 ± 0.5	6.9 ± 1.7	1.8 ± 0.6	2.7 ± 0.7	7.0 ± 0.2
**T/L**	0.1 ± 0.1	0.1 ± 0.0	0.2 ± 0.0	0.1 ± 0.1	0.2 ± 0.0	0.2 ± 0.0

Notes: Tissue uptakes of ^{64}Cu-Dox-immunoliposomes (5%PEG or 10%PEG) in A549 tumor xenograft-bearing BALB/c-nude mice (n = 3) at 1 h, 4 h, and 24 h post-i.v. injection expressed as %ID/g; Data (%ID/g) were presented as mean ± S.D. (n = 3).
